# A Case of Type 1 Cryoglobulinemia With Lymphoplasmacytic Lymphoma and Dry Gangrene

**DOI:** 10.7759/cureus.52659

**Published:** 2024-01-21

**Authors:** Abhinav K Rao, Fahim Syed, Diego Garrido, Charles S Holladay, Julia Saylors

**Affiliations:** 1 Department of Internal Medicine, Trident Medical Center, North Charleston, USA; 2 Department of General Surgery, Trident Medical Center, North Charleston, USA; 3 Department of Hematology and Oncology, Trident Medical Center, North Charleston, USA

**Keywords:** vascular occlusion, gangrene, plasmapharesis, chemotherapy response, chemotherapy

## Abstract

Lymphoplasmacytic lymphoma (LPL) is an uncommon condition, accounting for only 2% of all non-Hodgkin's lymphoma cases. Individuals with LPL face the risk of vascular blockage when associated with type I cryoglobulinemia, leading to related symptoms. Until now, no instances of LPL with dry gangrene have been documented. However, we present a rare case involving LPL accompanied by dry gangrene in both the right upper extremity (RUE) and left lower extremity (LLE). The patient was effectively managed using a combination of chemotherapy, steroids, plasmapheresis, and salvage surgery.

## Introduction

Lymphoplasmacytic lymphoma (LPL), sometimes referred to as Waldenstrom's macroglobulinemia, is a rare B-cell lymphoma. Symptoms arise due to organ infiltration by B cells, in addition to the properties of circulating pentameric immunoglobulin M (IgM) and associated vascular blockade, which is linked with underlying type I cryoglobulinemia. In addition to the typical B-cell symptoms, patients may present with symptoms related to vascular occlusion including digital ischemia, livedo reticularis, and skin necrosis [1]. Here, we present a rare case of a patient with LPL with dry gangrene. Prompt diagnosis, multidisciplinary collaboration, and multimodal treatment resulted in a favorable outcome for this patient.

## Case presentation

**Figure 1:**
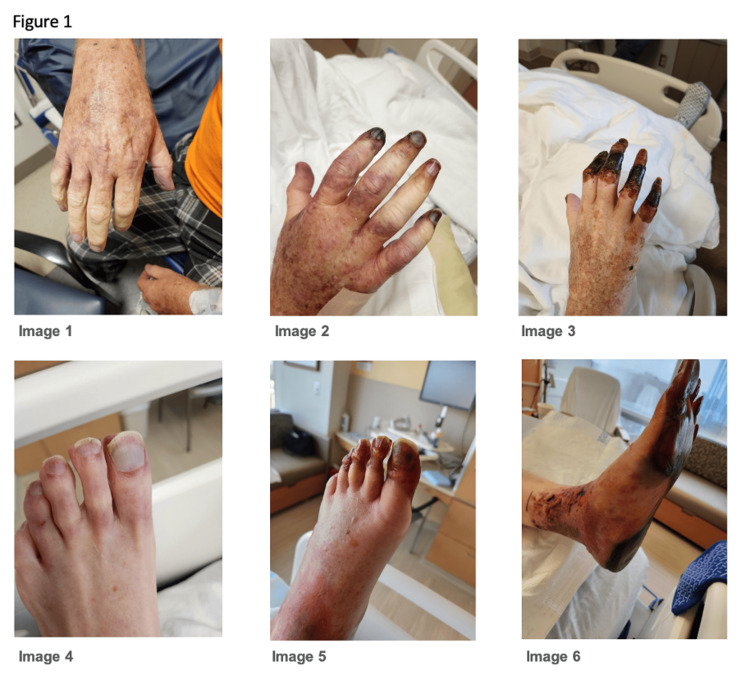
Extensive and progressive dry gangrenous necrosis of right upper extremity (RUE) and left lower extremity (LLE) Dry gangrene of RUE and LLE at initial hospitalization (Images 1 and 4), followed by worsening gangrene at 2 weeks (Images 2 and 5). Severe and extensive dry gangrenous necrosis on second hospitalization 1 month after initial insult (Images 3 and 6).

This patient is a 68-year-old male with a past medical history (PMH)of Raynaud’s phenomenon, prior pulmonary embolism taking apixaban** **2.5 BID, peripheral neuropathy, hypertension, asthma, and gastroesophageal reflux disease (GERD) who presented to an outside hospital with sudden onset right-hand pain, decreased sensation, and swelling of the right hand. At the time of presentation, the causes of his pulmonary embolism and peripheral neuropathy were undetermined. He also reported blue discoloration of the right hand for several days prior. He was diagnosed with the flu two days prior to presentation. He denied recent trauma and medication non-compliance. Vital signs were within normal limits. Physical examination revealed pallor in both hands. Radial pulses were symmetric and bounding. The right (R) hand was cooler than the left (L), and the sensation to tactile stimulation of the dorsal aspect of his R hand was decreased. The following day he developed similar discoloration of the left foot (Figure 1 - Images 1 and 4). His laboratory data were as follows: white blood cells (WBC) 8.1 cells/mcL, hemoglobin (Hgb) 12.7 gm/dL, hematocrit (Hct) 34.8 millions/mm3, platelets (Plt) 215 plts/uL, sodium (Na) 130 mmol/L, potassium (K) 4.0 mEq/L, chloride (Cl) 103 mEq/L, bicarbonate (CO2) 19 mmol/L, blood urea nitrogen (BUN) 14 mg/dL, creatinine (Cr) 1.18, mg/dL, glucose (Glu) 117 mg/dL, calcium (Ca) 8.1 mg/dl, total bilirubin 0.5 mg/dL, protein 5.9 g/dL, albumin 3.0 g/dL, aspartate aminotransferase (AST) 20 U/L, alanine aminotransferase (ALT) 19 U/L, alkaline phosphatase (Alk phos) 101 IU/L, international normalized ratio (INR) 1.59, partial thromboplastin time (PTT) 62.2 seconds. He was placed on a heparin drip due to concern for limb ischemia and received a dose of antibiotics in the emergency room.

A thromboembolic workup was initiated. An echocardiogram (ECHO) revealed mild aortic stenosis (AS) but otherwise no abnormality. Upper and lower extremity duplex ultrasound revealed patency with no evidence of thrombus. Computed tomography angiography (CTA) of the right upper extremity (RUE) revealed arterial patency. A hypercoagulability workup was initiated. Skin biopsy showed intraluminal vascular deposits of IgM cryoglobulins. Given the unclear etiology of his limb ischemia and IgM cryoglobulins in the skin biopsy, a bone marrow biopsy was pursued. His bone marrow biopsy revealed >30% lymphoplasmacytic infiltration exhibiting MYD88 L265P mutation, which was consistent with the diagnosis of lymphoplasmacytic lymphoma. Positron emission tomography-computed tomography (PET-CT) staging was performed which showed no active disease. A CT thorax did reveal 1.2 cm and 1.5 cm pulmonary and mediastinal lymph nodes, respectively, for which repeat imaging in 1 year was recommended. IgM paraprotein was confirmed on serum protein electrophoresis (SPEP) with a serum IgM was 150 at diagnosis with normal free light chain (FLC) and kappa/lambda (K/L) ratio. Beta-2 microglobulin (B2M) and serum viscosity were 2.61 mPA.s and 1.4 mPA.s, respectively. HIV, hepatitis B virus (HBV), and hepatitis C virus (HCV) were negative. Myelin-associated glycoprotein (MAG) and ganglioside (GM-1) autoantibody serologies were negative. He was treated with plasma exchange (PLEX) on presentation to reduce the circulating immune complex burden. After completing PLEX, he was treated with 1 cycle of bendamustine/rituximab (BR) with the plan to complete the rest of his cycles outpatient. He was also treated with prednisone with a rapid taper.

Of note, the patient developed acute blood loss anemia with retroperitoneal and scrotal hematoma during this admission while on anticoagulation. For this reason, he was treated with low-dose enoxaparin given the risk of bleeding with higher doses. He was then transitioned to apixaban 2.5 BID at discharge due to the high cost burden of enoxaparin. At the time of discharge, his gangrene remained localized with resolution in his cyanosis and pallor. He was also started on valaciclovir for mucosal ulcers that were found to be herpes simplex virus (HSV) positive.

One month later he presented to our hospital with 2 days of fatigue, fevers, and chills. Vital signs were within normal limits. His physical exam revealed extensive dry gangrene of his right fingers and left foot which was markedly worse from his prior hospitalization (Figure 1 - Images 2, 3, 5, 6). His laboratory data was unremarkable. He was started on vancomycin and piperacillin/tazobactam and anticoagulated with enoxaparin.

Further workup showed his repeat serum viscosity to be within normal limits (1.4 mPA.s). IgM was within the upper limit of normal (171 mg/dL). A CTA of the right upper extremity (RUE) with and without contrast was a somewhat limited study (portions of the right upper were clipped off the field of view) but showed normal-appearing arteries. CTA abdomen aorta with runoff was suggestive of median arcuate ligament impingement on the celiac trunk with no other significant peripheral artery disease. Ultrasound ankle/brachial indices (US ABI) of the L foot was 1.37 (R foot was 1.36). Blood cultures obtained on admission were without growth after 48 hours.

Due to patent arterial vasculature of his extremities, vascular surgery elected not to intervene. Although he had intact arterial flow, the foot was not salvageable due to necrosis of the forefoot as well as the skin and soft tissues of the plantar surface. Thus, general surgery offered a left below-the-knee amputation (BKA) to provide the best functional outcome, which the patient was agreeable to. A surgical specimen of the left lower extremity (LLE) showed ischemic gangrene of soft tissue and skin of the foot with no evidence of osteomyelitis, and severe atherosclerosis of the anterior and posterior tibial arteries, with viable resection margins. Due to gangrene in the right second, third, fourth, and fifth fingers, hand surgery was consulted, and recommended autoamputation of the R fingers to optimize wound healing. The patient was to continue amoxicillin-clavulanate for the duration to be determined until his fingers autoamputated and continue anticoagulation with apixaban** **2.5 mg BID. The patient was then discharged with the plan to complete his BR regimen with his outpatient hematologist and oncologist.

After discharge, the patient completed five of his six total cycles of his BR with minimal complications to date, with the plan to complete his last cycle at his next visit in 4 weeks. He developed mild nausea after his second cycle which resolved with ondansetron. He did have a mild infection of his right hand and completed a 10-day course of doxycycline prior to his third cycle. His fingers are yet to autoamputate. He has had good healing of the wound from his BKA and started using a prosthetic leg daily for up to 6 hours a day. He does experience phantom leg pain for which he is receiving pregabalin with some symptomatic improvement. His goal is to return to running. Otherwise, he is doing well overall with improving performance status. His most recent labs showed an absolute neutrophil count of 0.4, for which he was started on prophylactic levofloxacin 500 mg daily for 2 months and granulocyte-colony stimulating factor​​​​​​​ (GCSF)(pegfilgrastim​​​​​​​) with cycle 5 (C5). He continues on valaciclovir for his mucosal ulcers. His IgM improved to 200 prior to cycle 5, down from a peak of 312 prior to C3. He exhibits a good response to BR treatment with an appropriate decrease in his IgM levels.

## Discussion

Lymphoplasmacytic lymphoma (LPL), also referred to as Waldenstrom’s macroglobulinemia (WM), is rare, representing 2% of all cases of non-Hodgkin lymphoma. It has an incidence rate in the United States of about three cases per million people per year [1,2]. The clinical features of LPL arise secondary to organ infiltration by mature clonal B cells and IgM, in addition to specific immunologic and physio-chemical properties of circulating pentameric IgM. The former may lead to anemia, lymphadenopathy, and hepatosplenomegaly while the latter may cause cryoglobulinemia with subsequent hyperviscosity and symptoms related to vascular occlusion [3]. This patient had a history of Raynaud’s phenomenon and arthralgia which could have been early manifestations of type I cryoglobulinemia with the eventual development of digital ischemia and gangrene likely from cryoglobulinemia-mediated vascular occlusion. The patient had no symptoms of hyperviscosity, and his viscosity level was normal. His history of pulmonary embolism may have been due to circulating cryoglobulins within his pulmonary vessels. Additionally, he had symptomatic anemia and evidence of lymphadenopathy seen on his CT. His serum viscosity and IgM remained suppressed during his second hospitalization, likely due to the recent completion of his first cycle of treatment.

There have been few cases of reported gangrenous necrosis associated with LPL. One case reports a 57-year-old male with WM that presented gangrenous, hemorrhagic, bullous cellulitis associated with pseudomonas, who was in shock on arrival [4]. Another patient was found to have gangrenous cholecystitis associated with Waldenstrom’s macroglobulinemia [5]. Extensive limb gangrene in a patient with underlying type 1 cryoglobulinemia and multiple myeloma has also been reported [6].

This is one of few cases in which a patient developed dry gangrene due to underlying LPL and type 1 cryoglobulinemia. Though there was a concern for septic shock, this patient was afebrile, without tachycardia, hypotension, tachypnea, or leukocytosis on arrival. His blood cultures showed no growth after 5 days. Although he did receive plasmapheresis and was started on bendamustine/rituximab at an outside facility, the patient ultimately underwent L BKA due to a lack of salvageable tissue.

The prognostic score for LPL/WM is the International Prognostic Scoring System for Waldenstrom Macroglobulinemia (ISSWM). The following factors are used in determining patient prognosis: Age > 65, Hgb < 11.5, platelets < 100, B2M >3, serum IgM > 7g. Patients with 0-1 are low risk and have a median survival of 142 months. Those with a score of 2 factors or age alone are intermediate risk and have a median survival of 99 months. Finally, patients with 3 or more factors are at high risk correlating with a median survival of 43 months. This patient has two risk factors including Age >65 and Hgb < 11.5 which correlates with a score of 2 and intermediate risk [7].

With regard to treatment, his extensive limb ischemia (symptomatic cryoglobulinemia) warranted treatment with plasmapheresis during his first hospitalization. Red blood cell transfusion was avoided to prevent increases in serum viscosity. Though cascade filtration may offer a more selective method of macromolecule withdrawal, a study comparing it to plasma exchange showed it to be inferior in efficacy. Plasma exchange was more effective in decreasing plasma viscosity and IgM levels in patients with WM (plasma viscosity decreased by 48% in plasma exchange vs 26% in cascade filtration, IgM decreased by 42% in plasma exchange vs 27% cascade filtration) [4]. After completing the plasma exchange, the patient’s viscosity levels remained normal and his IgM remained suppressed.

To control malignant clonal cells, this patient underwent the standard treatment for symptomatic Waldenstrom’s macroglobulinemia, consisting of bendamustine plus rituximab (BR), which is usually given for four to six cycles [12], and a steroid taper. Rituximab has been shown to transiently increase serum immunoglobulin M level (IgM flare). This can be mitigated by either holding rituximab during the first cycle for patients with high IgM levels (>4000 mg/dL) or proceeding with plasma exchange prior to rituximab administration [8, 9]. The patient in this case did not have exceedingly high IgM levels and underwent plasmapheresis prior to chemotherapy, thereby reducing the risk of rituximab-induced hyperviscosity. Furthermore, he exhibited improvement in his IgM levels with treatment.

The decision to continue rituximab as maintenance therapy after induction therapy depends on the clinical scenario. The National Comprehensive Cancer Network (NCCN) recommends maintenance rituximab for patients who have partial or minor response to initial treatment. A randomized phase 3 trial reported that median progression-free survival (PFS) was longer with maintenance rituximab though statistical significance for this difference was not reached [10]. Furthermore, assessing the clinical response to induction therapy in this patient was difficult as irreversible gangrene occurred prior to chemotherapy administration. We would elect not to proceed with maintenance therapy in this patient given the lack of statistically significant benefit in studies done thus far, as well as, the additional toxicity, burden, and cost that comes with maintenance therapy [11, 12].

## Conclusions

We present here a rare case of dry gangrene in the setting of type 1 cryoglobulinemia with lymphoplasmacytic lymphoma (LPL). Though his limb ischemia was irreversible on presentation, prompt and appropriate treatment may help in preventing future episodes of limb ischemia in addition to prolonging median progression-free survival. He demonstrated good treatment response to bendamustine/rituximab as seen through reduction in his IgM levels and improvement in performance status. The patient’s preference in choosing to undergo L BKA may also reduce future sources of infection.
